# 5-year survival and rehospitalization due to stroke recurrence among patients with hemorrhagic or ischemic strokes in Singapore

**DOI:** 10.1186/1471-2377-13-133

**Published:** 2013-10-03

**Authors:** Yan Sun, Sze Haur Lee, Bee Hoon Heng, Vivien S Chin

**Affiliations:** 1 Department of Health Services & Outcomes Research, National Healthcare Group, Singapore, Singapore; 2 Department of Neurology, National Neuroscience Institute (NNI)-Tan Tock Seng Hospital (TTSH) Campus, Singapore, Singapore

**Keywords:** Stroke, Outcomes, Recurrence, Rehospitalization, Mortality, Singapore

## Abstract

**Background:**

Stroke is the 4th leading cause of death and 1st leading cause of disability in Singapore. However the information on long-term post stroke outcomes for Singaporean patients was limited. This study aimed to investigate the post stroke outcomes of 5-year survival and rehospitalization due to stroke recurrence for hemorrhagic and ischemic stroke patients in Singapore. The outcomes were stratified by age, ethnic group, gender and stroke types. The causes of death and stroke recurrence were also explored in the study.

**Methods:**

A multi-site retrospective cohort study. Patients admitted for stroke at any of the three hospitals in the National Healthcare Group of Singapore were included in the study. All study patients were followed up to 5 years. Kaplan-Meier was applied to study the time to first event, death or rehospitalization due to stroke recurrence. Cox proportional hazard model was applied to study the time to death with adjustment for stroke type, age, sex, ethnic group, and admission year. Cumulative incidence model with competing risk was applied for comparing the risks of rehospitalization due to stroke recurrence with death as the competing risk.

**Results:**

Totally 12,559 stroke patients were included in the study. Among them, 59.3% survived for 5 years; 18.4% were rehospitalized due to stroke recurrence in 5 years. The risk of stroke recurrence and mortality increased with age in all stroke types. Gender, ethnic group and admitting year were not significantly associated with the risk of mortality or stroke recurrence in hemorrhagic stroke. Male or Malay patient had higher risk of stroke recurrence and mortality in ischemic stroke. Hemorrhagic stroke had higher early mortality while ischemic stroke had higher recurrence and late mortality. The top cause of death among died stroke patients was cerebrovascular diseases, followed by pneumonia and ischemic heart diseases. The recurrent stroke was most likely to be the same type as the initial stroke among rehospitalized stroke patients.

**Conclusions:**

Five year post-stroke survival and rehospitalization due to stroke recurrence as well as their associations with patient demographics were studied for different stroke types in Singapore. Specific preventive strategies are needed to target the high risk groups to improve their long-term outcomes after acute stroke.

## Background

Stroke is one of the leading causes of mortality and morbidity in developed countries [1]. Stroke survivors often face ongoing high risks of mortality and stroke recurrence. Stroke patients and their families are concerned about the patient’s long-term post-stroke outcomes and therefore seek information that will enable them to make short and long-term plans and rational decisions [2]. Such information is also of interest to health care planners and providers [3,4]. The long-term survival and recurrence after stroke was widely studied, but the results were with great variation [5-16]. The reported relationships between patients’ demographics and the long-term post-stroke outcomes were also not consistent [17-25]. Majority of the studies were conducted in western population. Few comparisons have been made amongst Asian ethnic groups within the same geographical region.

Stroke is the fourth leading cause of death and hospitalization, as well as the first leading cause of long term disability in Singapore, especially in the elderly [26,27]. The prevalence rate of stroke in the Singapore population is about 3.7% amongst adults 50 years and above [28-30]. The prevalence rate is about 7.7% among adults aged 65 and above [28-30]. However, information regarding long-term post-stroke outcomes is scarce. Therefore this study aimed to firstly study the 5 year post-stroke outcomes of survival and rehospitalization due to recurrent stroke and their association with patient demographics in Singapore population; and secondly to study the causes of mortality for died stroke patients and the types of recurrent stroke for rehospitalized stroke patients.

## Methods

This is a multi-center retrospective cohort study. Patients who were admitted to any one of the three tertiary public hospitals in the National Healthcare Group of Singapore from year 2000 to year 2004, with a primary diagnosis of stroke (Ischemic Stroke [IS], Intracerebral Hemorrhage [ICH], or Subarachnoid Hemorrhage [SAH]), were included. The three study hospitals served about 40% of Singaporean population. Study patients’ data were extracted from the hospitals’ administrative databases, which store patients’ demographics, admission and discharge dates, diagnoses, movement and treatment during hospitalization. Stroke presentation was identified by the primary ICD-9-CM codes (International classification of diseases, ninth revision, clinical modification) of 430 (Subarachnoid Haemorrhage), 431 (Intracerebral Haemorrhage), 433 (Occlusion and stenosis of precerebral arteries), 434 (Occlusion of cerebral arteries), 436 (Acute, but ill-defined, cerebrovascular disease). Patients who were not Singapore citizens or permanent residents were excluded from the study as they were likely to be lost to follow-up. All study patients were followed up for 5 years from the admission day of their index hospitalization by tracing their electronic records. Patients who were readmitted to other hospitals other than the three study hospitals were lost to follow-up in this study.

Index hospitalization for stroke was defined as the first hospitalization during the study period with a principal diagnosis of stroke. Rehospitalisation for recurrent stroke was defined as the 2nd hospitalization with a principal diagnosis of stroke subsequent to the index hospitalization.

The variables studied were admitting year (2000–2004), age, gender, ethnicity (Chinese, Malay, Indian or others) and stroke subtypes (SAH, ICH or IS). The main outcomes of the study were 5-year survival and stroke recurrence, which were respectively defined as survival days from index admission date to date of death), and days from date of index admission to date of rehospitalization due to recurrent stroke.

Patients’ demographics of age, gender, ethnicity, and admitting year of the three stroke subtypes were compared using univariate tests. Kaplan-Meier analysis was applied to compare the cumulative incidence of death in 5 years among the patients of the three stroke subtypes. While cumulative incidence analysis with competing risk [31] was applied for hospitalization due to recurrent stroke.

Cox proportional model was applied to study both the unadjusted and adjusted relative hazard ratios of mortality in 5 years for all the stroke subtypes. The variables adjusted in the models were age, gender, ethnic group and admitting year. Fine and Gray competing risk model [32] was applied to estimate both the unadjusted and adjusted hazard ratios of hospitalization due to recurrent stroke in 5 years with death as a competing risk. The variables adjusted in the models were age, gender, ethnic group and admitting year.

The study was approved by the National Health Group’s research ethics committee, Domain Specific Review Boards (DSRB). All analyses were conducted using STATA version 11 (Stata Corporation, College Station, TX). Statistical significance was taken as *p*-value less than .05.

## Results

Totally there were 12,559 stroke patients included in the study. Of them, 9,554 (76.1%) were IS, 2,439 (19.4%) were ICH, and 565 (3.8%) were SAH. Overall, 58.3% of the patients survived after 5 years, 15.7% of them were rehospitalized due to recurrent stroke. 55.2% of SAH, 55.4% of ICH, and 59.2% of IS patients survived for 5 years. 4.8% of SAH, 8.9% of ICH, and 14.6% of IS patients were re-hospitalized due to recurrent stroke in the 5 years.

Table 1 shows patient characteristics of age, gender, ethnic group, admitting year, the proportions of mortality and stroke recurrence in all the three types of stroke patients. The patients were predominantly Chinese (81.2%), Malay (10.6%), Indian (5.5%), and other ethnic groups (3.7%) made up the rest of the sample. The ethnic distribution within the national population at the start of the study was 76.8% Chinese, 13.9% Malay, 7.9% Indian and 1.4% other ethnic groups [11]. There were more males than females (54.0% vs. 46.0%), *p* < .001. The mean age was 76.3 (SD = 13.8) and the median age was 77.4 years old. Totally, 58.3% of stroke patients survived longer than 5 years. 13.0% of stroke patients were reshospitalized due to recurrent stroke in 5 years. Overall, SAH patients (mean: 70.5; median: 72.0) were younger than ICH patients (mean: 74.6; median: 73.5); and ICH patients were younger than IS patients (mean: 78.4; median: 77.4). Female were more likely to get SAH; but less likely to get ICH or IS compared with male. The ethnic distribution in each stroke type was similar. More IS survived for 5 years than ICH and SAH patients. However, a smaller proportion of ICH and SAH patients were rehospitalized due to recurrent stroke (Table 1).

**Table 1:** Characteristics of stroke patients

**Variables**		**Total (n = 12,559)**	**ICH (n = 2439)**	**IS (n = 9555)**	**SAH (n = 565)**	**p-value**
Age (Years)	Median	77.4	74.6	78.4	72.0	<0.001
Gender	Female (%)	5,778 (46.0)	1,036 (42.5)	4,421 (46.3)	321 (56.8)	<0.001
	Male (%)	6,780 (54.0)	1,403 (57.5)	5,133 (53.7)	244 (43.2)	
Ethnic group	Chinese (%)	10,202 (81.2)	2,000 (82.0)	7,750 (81.1)	452 (80)	<0.001
	Malay (%)	1,326 (10.6)	283 (11.6)	975 (10.2)	68 (12)	
	Indian (%)	695 (5.5)	88 (3.6)	583 (6.1)	24 (4.2)	
	Others (%)	335 (2.7)	68 (2.8)	246 (2.6)	21 (3.7)	
Admitting Year	2000	2649 (21.1)	583 (23.9)	1946 (20.4)	120 (21.2)	<0.001
	2001	2702 (21.5)	493 (20.2)	2089 (21.9)	120 (21.2)	
	2002	2750 (21.9)	529 (21.7)	2112 (22.1)	109 (19.3)	
	2003	2050 (16.3)	380 (15.6)	1566 (16.4)	104 (18.4)	
	2004	2408 (19.2)	454 (18.6)	1842 (19.3)	112 (19.8)	
Death	No (%)	7,320 (58.3)	1,351 (55.4)	5,657 (59.2)	312 (55.2)	<0.001
	Yes (%)	5,238 (41.7)	1,088 (44.6)	3,897 (40.8)	253 (44.8)	
Stroke recurrence	No (%)	10,922 (87.0)	2,221 (91.1)	8,163 (85.4)	538 (95.2)	<0.001
	Yes (%)	1,636 (13.0)	218 (8.9)	1391 (14.6)	27 (4.8)	

**Figure 1:**
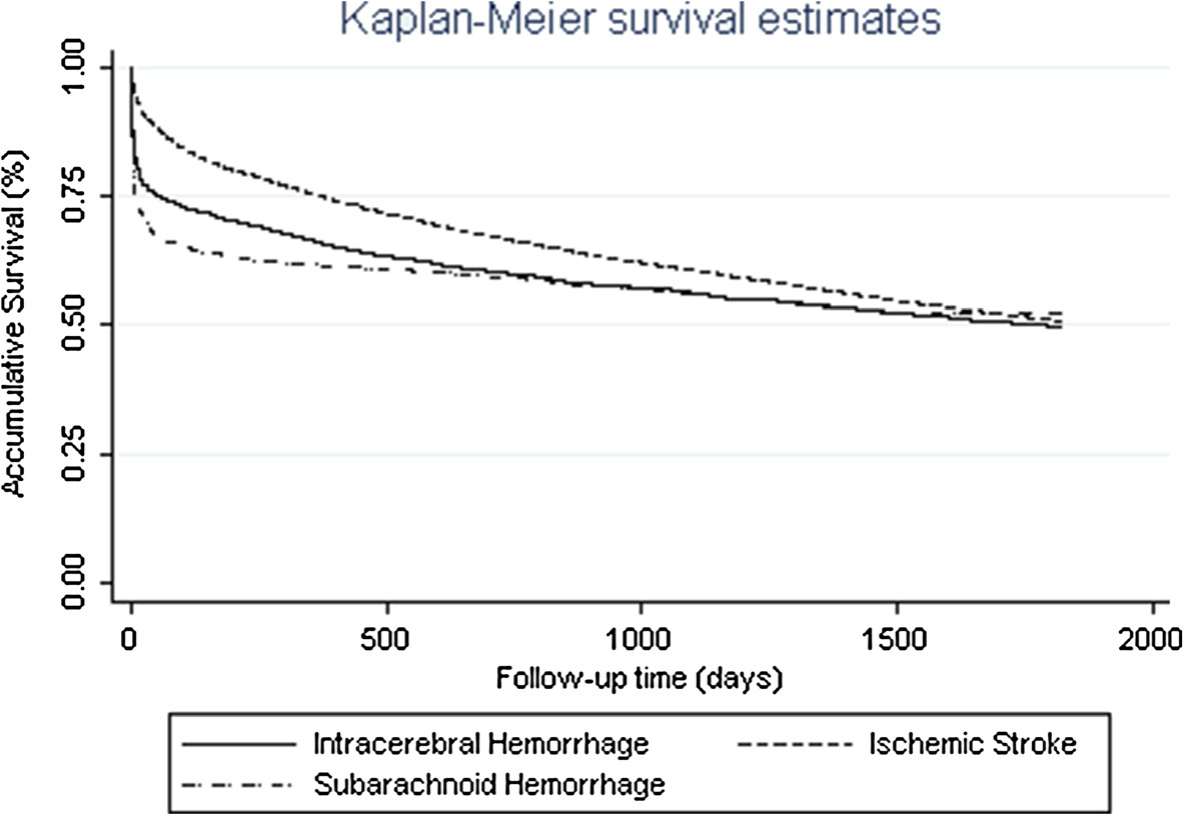
5-year survival by stroke subtype by Kaplan-Meier analysis.

The survival curves of the three types of stroke patients were compared using Kaplan-Meier analysis. Ischemic stroke patients survived significantly longer than hemorrhagic patients (ICH then followed by SAH). The average survival time for IS patients were 3.6 years; while the time for ICH and SAH were 3.0 years and 2.8 years, respectively. For SAH patients, once they survived the first month, they were more likely to survive the following 5 years. For ICH patients, they died fast at the beginning, and they also died fast in the following years. While for IS patient, they had high survival chance at the beginning, but they were still at high risk of death in the following 5 years. The log rank test suggested that the survival functions of the three types of stroke were significantly unequal (chi-square statistics = 149.5, and p < 0.001) (Figure 1).

**Figure 2:**
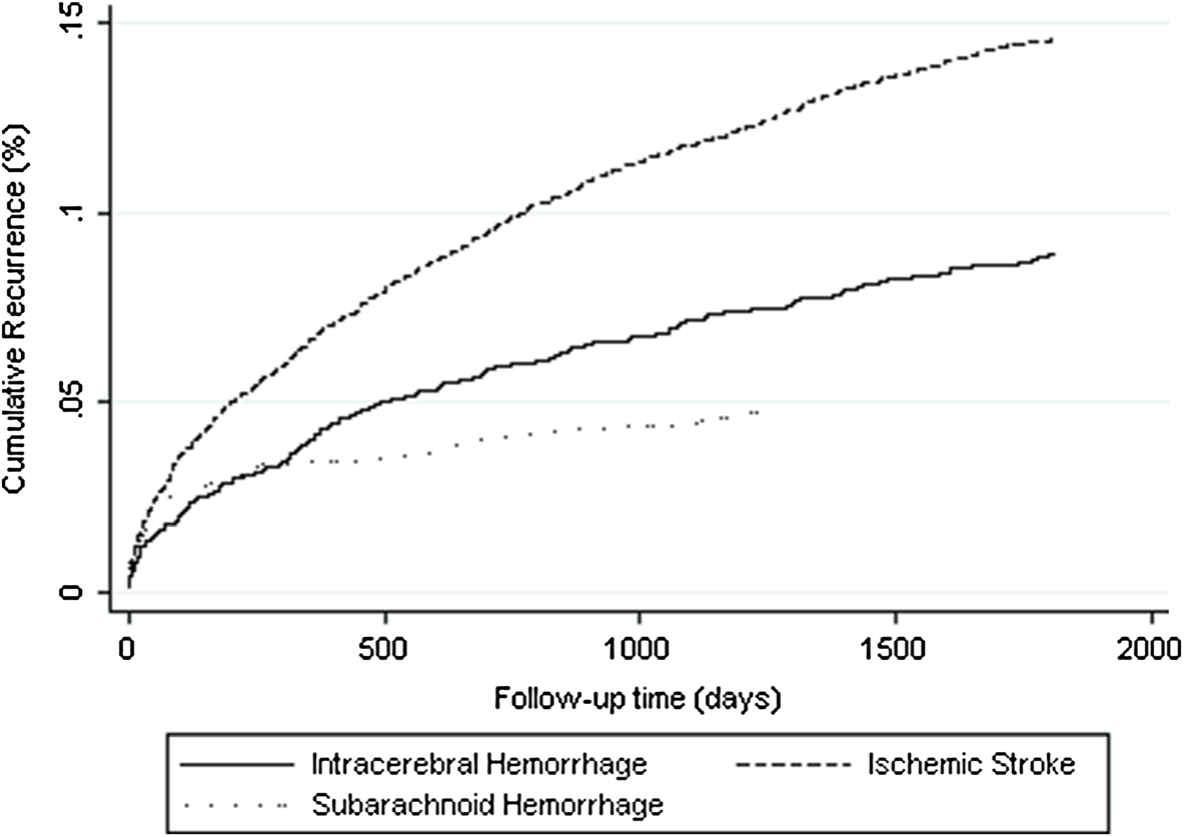
5-year rehospitalization due to stroke recurrence by stroke subtype by cumulative incidence analysis with competing risk.

The risks of hospitalization due to stroke recurrence over the 5 years among the three types of stroke patients were compared using cumulative incidence analysis with death as competing risk. Hemorrhagic stroke patients were more likely to be hospitalized than ischemic patients. The Gary test suggested that the survival functions of the three types of stroke were significantly unequal (chi-square statistics = 96.5, and p < 0.001) (Figure 2).

The unadjusted odds ratios of death in 5 years in ICH and SAH group were 1.4 (95%CI: 1.3-1.5) and 1.6 (95%CI: 1.4-1.8) respectively, as compared with IS group. The adjusted associations between the risk of death and the factors of admitting year, age, gender and ethnic group in the three stroke categories were estimated using cox regression. In all three stroke types, after adjusting for other factors, elder age increased the risk of mortality. But stroke mortality occurred across all age groups. Among stroke patients younger than 45 years, 10.1% of IS patients, 23.2% of ICH patients, and 13.2% of SAH patients died in 5 years. For IS patients, the mortality rates decreased with admitting year; being male or in Malay or other ethnic group were at a higher adjusted relative risk of death as compared with Chinese. While for ICH or SAH patients, the risk of mortality in 5 years wasn’t affected by admitting year, gender, or ethnic group (Table 2).

**Table 2:** Risk ratio of death by cox regression

	**IS**		**ICH**		**SAH**	
	Crude ratio	Adjusted ratio	Crude ratio	Adjusted ratio	Crude ratio	Adjusted ratio
Age	**1.04 (1.03–1.04)**	**1.04 (1.04–1.04)**	**1.03 (1.02–1.03)**	**1.03 (1.02–1.03)**	**1.03 (1.02–1.04)**	**1.03 (1.02–1.04)**
Ethnic group [Chinese]	1	1	1	1	1	1
Malay	**1.18 (1.07-1.30)**	**1.33 (1.20-1.47)**	1.03 (0.86-1.24)	1.09 (0.91-1.31)	1.07 (0.74-1.55)	1.09 (0.75-1.58)
Indian	0.99 (0.87-1.14)	1.13 (0.99-1.30)	0.77 (0.54-1.09)	0.78 (0.55-1.12)	0.86 (0.44-1.68)	1.03 (0.52-2.01)
Other	1.19 (.99-1.44)	**1.25 (1.04–1.51)**	1.35 (0.97-1.87)	1.38 (0.99-1.91)	1.30 (0.71-2.39)	1.34 (0.73-2.45)
Gender [Female]	1	1	1	1	1	1
Male	**0.92 (0.87-0.98)**	**1.10 (1.03 – 1.18)**	0.99 (0.88-1.11)	1.12 (0.99-1.26)	0.94 (0.74-1.21)	1.11 (0.86-1.43)
Admitting year	**0.97 (0.94-0.99)**	0.99 (0.97-1.01)	1.02 (0.98-1.06)	1.04 (0.99-1.08)	0.93 (0.86-1.01)	0.96 (0.88-1.04)

Reference group: [].

Considering death as the competing risk, the crude odds ratios of hospitalization due to stroke recurrence were 1.1 (95%CI: 1.1-1.2) and 1.1 (95%CI: 0.9-1.3) in ICH and SAH groups respectively, as compared with IS patients. The adjusted associations between the risk of hospitalization due to stroke recurrence and the factors of admitting year, age, gender and ethnic group in the three stroke category were estimated by Fine and Gary model. Older patients had higher risk of rehospitalization in all three types of stroke. Ethnic group, gender and admitting year didn’t affect the risk of rehospitalization in the hemorrhagic stroke categories (ICH and SAH). Among IS patients, discharged in earlier years, males, Malays and other ethnic groups had higher relative risk of stroke recurrence (Table 3).

**Table 3:** Risk ratio of stroke recurrence by Fine-Gary model

	**IS**		**ICH**		**SAH**	
	Crude ratio	Adjusted ratio	Crude ratio	Adjusted ratio	Crude ratio	Adjusted ratio
Age	1.03 (1.03–1.03)	1.03 (1.03-1.03)	1.02 (1.02–1.03)	1.02 (1.02-1.03)	1.03 (1.02–1.04)	1.03 (1.02-1.04)
Ethnic group						
[Chinese]	1	1	1	1	1	1
Malay	1.09 (0.99–1.20)	1.19 (1.08-1.31)	1.05 (.89–1.24)	1.09 (0.92-1.29)	1.11 (.79–1.57)	1.11 (0.79-1.57)
Indian	.98 (.87–1.11)	1.08 (0.96-1.22)	0.84 (.62–1.13)	0.85 (0.62-1.16)	1.01 (.54–1.88)	1.20 (0.67-2.16)
Other	1.20 (1.01–1.42)	1.24 (1.05-1.46)	1.28 (.93–1.76)	1.30 (0.94-1.80)	1.35 (.76–2.39)	1.32 (0.79-2.22)
Gender [Female]	1	1	1	1	1	1
Male	.96 (.91–1.02)	1.10 (1.04-1.17)	0.97 (.87–1.09)	1.07 (0.96-1.20)	0.96 (0.75–1.22)	1.12 (0.87-1.42)
Admitting year	0.96 (0.93-1.01)	0.97 (0.93-1.01)	0.96 (0.87-1.05)	0.97 (0.87-1.05)	1.02 (0.79-1.33)	1.04 (0.80-1.36)

Reference group: [].

**Figure 3:**
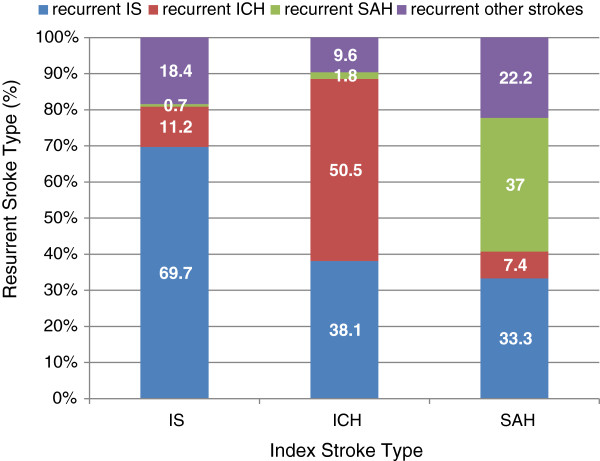
Recurrent stroke types for patients admitted with the three stoke types.

We also studied the relationship between the first stroke and the recurrent stroke. Figure 3 shows the distribution of the stroke types identified in recurrent stroke hospitalization with respect to the initial stroke type. The recurrent stroke was most likely to be the same type as the initial stroke among the rehospitalized stroke patients. About 70% of recurrent stroke were still IS stroke among patients with initial IS. About 60% of recurrent stroke were still ICH among patients with initial ICH. About 40% of recurrent stroke were still SAH stroke among patients with initial SAH. The percent distribution of the recurrent stroke types were significantly different among the IS, ICH and SAH patients (chi-square statistics = 21.9, and p < 0.001) (Figure 3).

**Figure 4:**
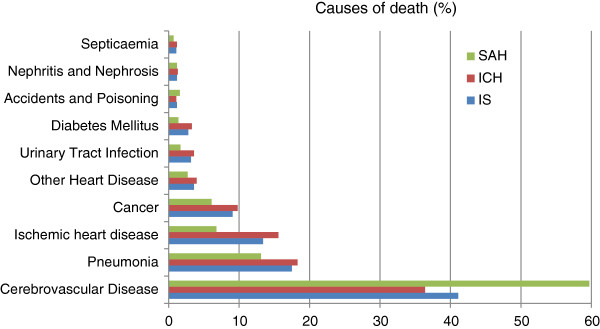
The top 10 causes of death for patients admitted with the three stroke types.

Cerebrovascular disease was the leading cause of death, followed by pneumonia, ischemic heart disease and cancer, for all types of stroke patients. Other causes of death among stroke patients included other heart disease, UTI, diabetes mellitus, accidents, poisoning and violence, nephritis, nephrotic syndrome and nephrosis, as well as septicaemia. 36.4% of death for IS patients were caused by cerebrovascular disease, while the proportion was 60% for ICH patients and 70% for SAH patients. The causes of death were significantly different among died IS, ICH and SAH patients (chi-square statistics = 39. 5, and p < 0.001) (Figure 4).

## Discussion

In our study, 15.7% of stroke patients were rehospitalized due to recurrent stroke, and 58.3% of the patients survived after 5 years. A wide range of survival (29.0%-60.3%) and recurrent probabilities (9.4%-23.8%) have been reported cross different population [7-16], which could in part be explained by differences in study population, stroke subtypes, stroke severity, first-ever or recurrent stroke. Our results were within the reported range. IS patients had the highest survival rate, followed by ICH and then SAH patients in this study. Compared with IS and SAH patients, ICH patients were more likely to be hospitalized due to stroke recurrence after considering death as the competing risk. If competing risk of death was not considered, IS had highest risk of stroke recurrence, followed by ICH and SAH, which were tally with the reported findings [7-16]. Therefore adjustment of competing risk of death is crucial for studying the risk of stroke recurrence.

It is not surprise that older patients had higher risks of mortality and stroke recurrence in all stroke categories in this study. Gender did not significantly influence outcomes except IS in this study. After adjusting for admitting year, age, and ethnic group, females had a lower relative risk of mortality than males among IS patients. According to the National Health Survey conducted in year 2004 in Singapore [33], males had higher prevalence of DM, HTN, total cholesterol, and smoking compared to females. This may help explain the gender difference to some extend in this study. How gender affects the post-stroke survival was inconclusive based on published studies [17-21]. This discrepancy might be caused by sample size, stroke subtype, stroke severity, and confounders adjusted in the studies.

In our study, ethnic group didn’t influence the 5 year risk of mortality and stroke recurrence except for IS patients. There was an increased risk of mortality and stroke recurrence in Malays and other ethnic groups as compared with Chinese among IS patients. This trend may be partially explained by the higher load of atherosclerotic risk factors among them. Malays had higher prevalence of DM, lipids, and smoking compared to Chinese according to the National Health Survey 2004 [33]. Differences in outcomes for various ethnic groups have also been reported in other studies [22-25].

We also examined the causes of death for patients with different subtypes of stroke. A large proportion of deaths were related to stroke complications. Complications of stroke can be classified in the cerebral and extra cerebral [34]. Extra cerebral complications include pneumonia and pneumonitis, acute hypertensive response, venous thromboembolism, cardiac complications, urinary tract infections and decubitus ulcers [34]. The leading causes of death in this study were cerebrovascular disease, followed by pneumonia, ischemic heart disease and cancer. The leading cause of death for women was cerebrovascular disease whilst pneumonia for men. Hemorrhagic stroke patients were more likely to die from cerebrovascular disease compared to IS patients.

In sum, older stroke patients have a higher risk of worse outcomes. Ethnic differences are seen in the outcomes of stroke depending on the type of stroke. Females have better outcomes than males only for IS patients. The stroke recorded during the subsequent hospitalization is also more likely to be of the same type as the initial stroke identified. The most common cause of death post-stroke is cerebrovascular diseases in all the stroke patients regardless the subtype of stroke.

The main strengths of this study are that we have studied about 40% of the stroke population across multi-hospitals in Singapore and have obtained complete five-year follow-up. This is the largest study of survival and recurrent stroke to date in Singapore. In addition we have used methods of survival analysis with competing risk that allowed us to estimate the risks of recurrent stroke and death simultaneously. Competing risk model is more appropriate for a condition such as stroke which is associated with a high mortality.

There are a few limitations to our study. First, we have used ICD diagnosis codes in the administrative databases to identify our study patients. Internal audit studies have shown that the coding accuracy of primary diagnoses were above 96%. However, the accuracy of coding is likely to vary with disease complexity or differ in institutions. Second, stroke patients who readmitted to other hospitals than the three study hospitals were not captured. Therefore the readmission rate due to stroke recurrence might be under-estimated in this study. However, based on internal audit studies, among all the readmitted patients in one year after discharge, less than 6% were readmitted to other hospitals. This rate may vary for patients with different diseases. Third, our risk adjustment may be inadequate because of lack of clinical details, for example the severity of stroke. Lastly, we couldn’t differentiate patients of first ever stroke or the recurrent stroke based on their index admission. The 5-year survival and recurrent rates might be very different for these two kinds of patients.

## Conclusions

Five year post-stroke survival and rehospitalization due to stroke recurrence as well as their associations with patient demographics were studied for different stroke types in Singapore. The causes for post-stroke mortality and the recurrent stroke types were also studied. Specific preventive strategies are needed to target the high risk groups to improve their long-term outcomes after acute stroke.

## Competing interests

The author(s) declare that they have no competing interests. All data, results and copyrights of this research are belonging to the National Healthcare Group of Singapore. There are no any other direct or indirect sponsors of this study.

## Authors’ contributions

SY designed the study, did the statistical analysis and wrote the first draft. LSH and BHH made substantial contributions to study design, the discussion and explanation of results. CVS did some of the data analysis and contributed to manuscript drafting. All authors have read and approved of the content of the final submitted manuscript. The corresponding author holds the final responsibility for the decision to submit for publication. All authors read and approved the final manuscript.

## Pre-publication history

The pre-publication history for this paper can be accessed here:

http://www.biomedcentral.com/1471-2377/13/133/prepub
